# Combination of Quantitative MRI Fat Fraction and Texture Analysis to Evaluate Spastic Muscles of Children With Cerebral Palsy

**DOI:** 10.3389/fneur.2021.633808

**Published:** 2021-03-22

**Authors:** Tugba Akinci D'Antonoli, Francesco Santini, Xeni Deligianni, Meritxell Garcia Alzamora, Erich Rutz, Oliver Bieri, Reinald Brunner, Claudia Weidensteiner

**Affiliations:** ^1^Department of Pediatric Radiology, University Children's Hospital Basel, Basel, Switzerland; ^2^Department of Radiology, University Hospital of Basel, Basel, Switzerland; ^3^Division of Radiological Physics, Department of Radiology, University Hospital of Basel, Basel, Switzerland; ^4^Department of Biomedical Engineering, University of Basel, Basel, Switzerland; ^5^Division of Diagnostic and Interventional Neuroradiology, University Hospital of Basel, Basel, Switzerland; ^6^Pediatric Orthopedic Department, Murdoch Children's Research Institute, The Royal Children's Hospital, MCRI the University of Melbourne, Melbourne, VIC, Australia; ^7^Faculty of Medicine, The University of Basel, Basel, Switzerland; ^8^University Children's Hospital Basel, Basel, Switzerland; ^9^Department of Orthopedic Surgery, University Children's Hospital Basel, Basel, Switzerland

**Keywords:** cerebral palsy, pediatric imaging, dixon imaging, intramuscular fat, magnetic resonance imaging, radiomics analysis, texture analysis

## Abstract

**Background:** Cerebral palsy (CP) is the most common cause of physical disability in childhood. Muscle pathologies occur due to spasticity and contractures; therefore, diagnostic imaging to detect pathologies is often required. Imaging has been used to assess torsion or estimate muscle volume, but additional methods for characterizing muscle composition have not thoroughly been investigated. MRI fat fraction (FF) measurement can quantify muscle fat and is often a part of standard imaging in neuromuscular dystrophies. To date, FF has been used to quantify muscle fat and assess function in CP. In this study, we aimed to utilize a radiomics and FF analysis along with the combination of both methods to differentiate affected muscles from healthy ones.

**Materials and Methods:** A total of 9 patients (age range 8–15 years) with CP and 12 healthy controls (age range 9–16 years) were prospectively enrolled (2018–2020) after ethics committee approval. Multi-echo Dixon acquisition of the calf muscles was used for FF calculation. The images of the second echo (TE = 2.87 ms) were used for feature extraction from the soleus, gastrocnemius medialis, and gastrocnemius lateralis muscles. The least absolute shrinkage and selection operator (LASSO) regression was employed for feature selection. RM, FF model (FFM), and combined model (CM) were built for each calf muscle. The receiver operating characteristic (ROC) curve and their respective area under the curve (AUC) values were used to evaluate model performance.

**Results:** In total, the affected legs of 9 CP patients and the dominant legs of 12 healthy controls were analyzed. The performance of RM for soleus, gastrocnemius medialis, and gastrocnemius lateralis (AUC 0.92, 0.92, 0.82, respectively) was better than the FFM (AUC 0.88, 0.85, 0.69, respectively). The combination of both models always had a better performance than RM or FFM (AUC 0.95, 0.93, 0.83). FF was higher in the patient group (FF_S_ 9.1%, FF_GM_ 8.5%, and FF_GL_ 10.2%) than control group (FF_S_ 3.3%, FF_GM_ 4.1%, FF_GL_ 6.6%).

**Conclusion:** The combination of MRI quantitative fat fraction analysis and texture analysis of muscles is a promising tool to evaluate muscle pathologies due to CP in a non-invasive manner.

## Introduction

Cerebral palsy (CP) is the most common cause of physical disability in childhood, caused by brain injury during the antenatal or early postnatal period (1). Although primary damage occurs in the central nervous system, clinical symptoms are mostly associated with the peripheral neuromuscular system, particularly with skeletal muscles (2). The severity of the clinical manifestations depends on the degree of the injury, ranging from mild movement disorder to severe functional limitation (2). Muscle pathologies occur due to spasticity and contractures, and so far, those pathologies are assessed by either clinical scoring systems, e.g., modified Ashworth scale (MAS), or invasive procedures, e.g., biopsies (2). Imaging has been used to assess torsion or estimate muscle volume (3, 4), but additional methods for characterizing muscle composition have not thoroughly been investigated.

Quantitative magnetic resonance imaging (MRI) is a promising non-invasive imaging modality to assess pathologic changes in muscles. Particularly in neuromuscular muscle diseases, quantitative MRI methods have already become standard for disease monitoring (5–8). Among these quantification methods, fat fraction (FF) measurement is commonly employed to determine fatty infiltration in a muscle, providing insights into function and pathophysiology (6, 7, 9, 10). Most of the quantification methods are based on a mean value calculation within a region of interest (ROI); however, mean values cannot entirely capture the heterogeneity or dynamic variations within the ROI and, therefore, will not show a robust correlation with tissue characteristics (11, 12). So far, FF analysis has been rarely employed to evaluate CP patients (13, 14).

Texture analysis (also called radiomics) is an advanced technique that aims to extract quantitative parameters from diagnostic images to discover the relationship between imaging features and the underlying biological information (15). To date, radiomics analysis has been mostly applied in the field of oncology—including but not limited to gene-expression pattern prediction (16), lesion characteristic discrimination (17), and treatment outcome prediction (18). Radiomics analysis of skeletal muscles recently gained more attention with the increased understanding of the relationship between muscle texture changes and disease pathophysiology (19, 20). Up to now, few studies have focused on radiomics analysis of skeletal muscles, and most of them were either animal studies or in healthy populations (21–27). The potential of the texture analysis of pathologic muscles in human subjects has rarely been explored (28, 29). To our knowledge, our study is the first to employ muscle texture analysis along with FF measurement in children with CP.

In this study, we aimed to employ radiomics and FF analysis along with the combination of both methods to differentiate pathologic muscles from healthy ones in children with CP and healthy controls and compare our RM with FFM and with a combination of both models.

## Materials and Methods

### Study Population

A total of nine patients (median age 11.5 years) with CP and 12 age-/height-/weight-matched healthy controls (median age 11.1 years) were prospectively enrolled between 2018 and 2020 after ethics committee approval (Table 1). All patients were into consideration for corrective surgery. They had fixed contractures with a functional component contributing to equinus gait. Six patients were diagnosed with unilateral spastic hemiparesis and 3 with spastic diparesis. At the time of the study, the MAS ranged between 0 and 2; only two hemiparetic patients had MAS of 0. The Gross Motor Function Classification System (GMFCS) level was mostly I, only two patients had level II, and another had level III motor function impediment. Passive range of motion (ROM) and manual muscle testing (MMT) of the knee joint was reported in Table 2. Informed consent was obtained from the parents of the participants and, additionally, from the 12 years old or older participants at the time of examination. Exclusion criteria were a history of surgery on the affected limb(s), claustrophobia, and failing to follow instructions during the acquisition.

**Table 1:** All study participant demographics.

**Parameter**	**Control group (*n* = 12)**	**Cerebral palsy group (*n* = 9)**	**Fisher's exact test *P* value**
Age	11.1 (9.6–13.7)	11.5 (10.6–12.0)	0.730
Sex			0.061
Female, *n* (%) Male, *n* (%)	6 (50%) 6 (50%)	1 (11%) 8 (89%)	
Height	140.0 (134.0–162.5)	146.0 (135.0–151.0)	0.634
Weight	32.35 (28.15–49.25)	32.7 (26.6–44.6)	0.822
BMI	16.4 (15.7–17.3)	16.1 (14.6–18.3)	0.861

*Unless otherwise specified, data are medians and interquartile ranges. BMI, body mass index*.

**Table 2:** Patient characteristics, results of the clinical examination, passive range of motion, manual muscle testing, and MRI fat fraction.

**Patient**	**Patient Characteristics**				**Spasticity**^**‡**^			**PROM**		**MMT**^**∧**^		Fat Fraction^*^		
	**BMI percentile^**†**^**	**CP**	**GMFCS**	**More affected side**	**PF (at 90^**°**^ KF)**	**PF (at KE)**	**KF**	**DF (at KE)**	**KE (at HE)**	**PF**	**KF**	**S**	**GM**	**GL**
1	5	Unilateral	I	Right	1+	1+	0	−10°	0°	2+	5	6.8	5.5	16.7
2	95	Unilateral	I	Right	0	1	0	−20°	−15°	2+	4	24.4	16.5	16.5
3	23	Unilateral	I	Right	1+	1	0	15°	0°	2+	5	7.1	7.7	19.2
4	49	Bilateral	I	Left	1	1	0	−10°	−20°	2+	4	9.4	8.1	5.9
5	3	Bilateral	II	Left	1+	4	2	−30°	−5°	3	4	13.6	13.5	11.7
6	4	Bilateral	III	Left	1	1	1	−5°	−10°	3+	3	6	8.1	7.4
7	71	Unilateral	II	Left	2	2	0	10°	0°	2	4	5.1	5	4.7
8	61	Unilateral	I	Right	1	1	0	0°	10°	2+	5	4.1	3.6	4.3
9	5	Unilateral	I	Left	0	0	0	10°	5°	2+	4	3.3	8.2	5.1

*PROM, passive range of motion; MMT, manual muscle testing; BMI, body mass index; CP, cerebral palsy; GMFCS, Gross Motor Function Classification System; PF, plantarflexor muscles; KF, knee flexion; KE, knee extension; DF, dorsiflexion; HE, hip extension; S, Soleus; GM, Gastrocnemius medialis; GL, Gastrocnemius lateralis*.

† *BMI percentile: underweight <5; 5 ≤ normal weight <85; 85 ≤ overweight <95; 95 ≤ obesity*.

† *Modified Ashworth Scale*.

∧ *Medical Research Council scale*.

* *Fat fraction percentage in calf muscles of the affected legs of unilateral and more affected legs of the bilateral CP patients*.

### Image Acquisition

The same scanner and the same acquisition parameters were used throughout the study. MRI exams were performed with a 3T whole-body scanner (Siemens Prisma, Siemens Healthineers, Erlangen, Germany). The patients and healthy controls were positioned supine on the patient table, and the lower extremity was restrained with straps at a comfortable resting angle. A Siemens 18-element-body array coil was placed on the lower leg. A three-dimensional (3D) multi-echo gradient-echo (Dixon) sequence was used to reconstruct fat-only and water-only images: 6 echoes: echo times (TEs) 1.41/2.87/4.33/5.79/7.25/8.71 ms, repetition time (TR) = 20 ms, voxel size 1.1 × 1.1 × 3.0 mm^3^, reconstructed matrix = 320 × 190 × 96, flip angle = 12°, acceleration factor 2, acquisition time 4 min 49 s (30). Images were acquired without contrast material and without anesthesia or sedation. Children were offered the possibility of visual or audio entertainment during the examination to improve compliance, and in case of suboptimal image quality, the corresponding acquisition was repeated.

### Image Segmentation and Analysis

**Figure 1:**
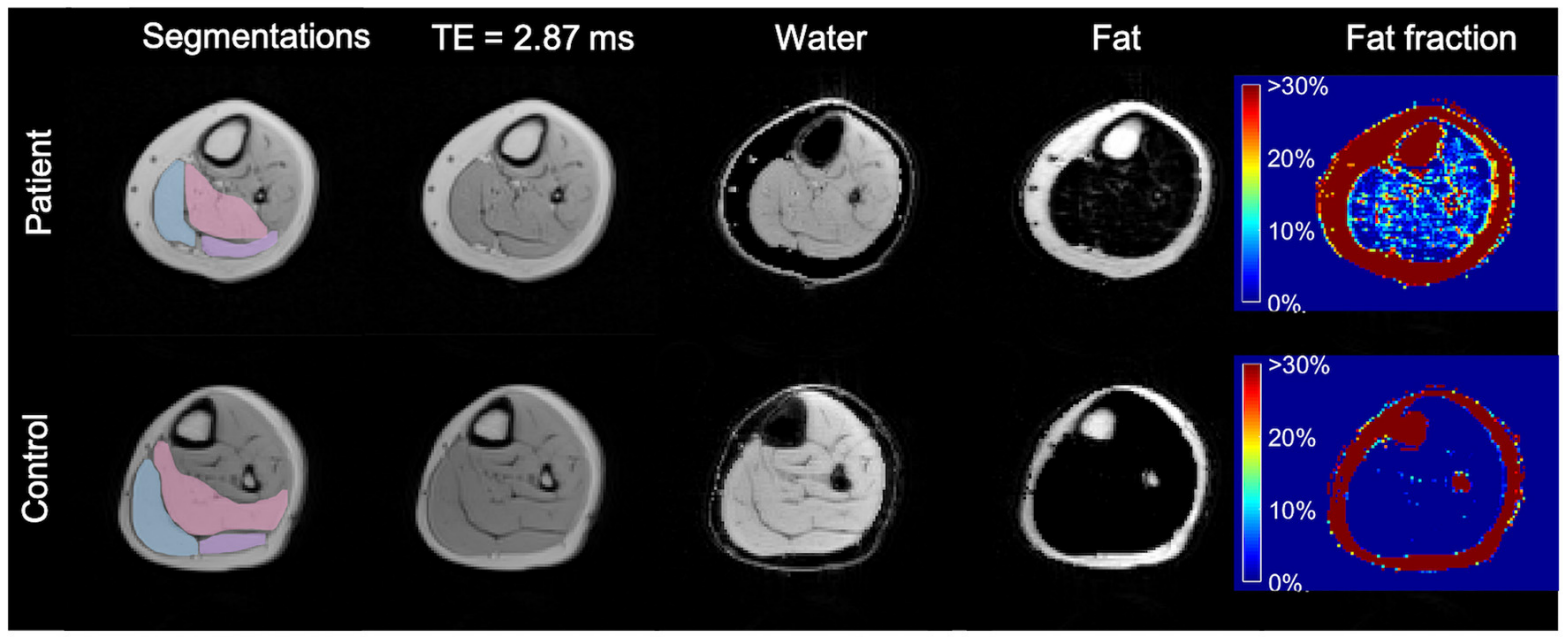
Axial MR images of the more affected calf of a patient (diparetic, boy, 11 years) and the dominant calf of healthy control (a typically developing 11 years boy with similar BMI). First column: segmented ROIs for soleus (pink), gastrocnemius medialis (light blue), and gastrocnemius lateralis (lilac); second column: 2nd echo image from the Dixon data set; third column: water-only image calculated from the Dixon dataset; fourth column: fat-only image calculated from the Dixon dataset; fifth column: fat fraction map ranging from 0 to 100% calculated from the Dixon dataset, showing a higher fat fraction in the CP patient. TE, Echo Time.

The images of the second echo (TE = 2.87 ms) from the multi-echo gradient-echo Dixon acquisition were used for the muscle segmentation. An in-house developed segmentation tool was employed for manual contour delineation. An experienced radiologist (T.A.D.) was responsible for all segmentations. The entire volumes of the calf muscles—soleus (S), gastrocnemius medialis (GM), and gastrocnemius lateralis (GL)—were segmented for both legs (Figure 1). Segmentations were then reviewed by one of the authors (C.W.) to check for errors.

All ROI margins were eroded by one voxel to reduce partial volume effects from adjacent adipose tissue and to prevent possible inadvertent overlaps between ROIs.

Water-only images, fat-only images, and fat fraction maps (defined as the signal intensity of the fat-only images divided by the sum of the signal intensities of fat-only and water-only images) were calculated online by the scanner software for all calf muscles bilaterally (30) (Figure 1).

Texture analysis was applied to the second echo images of the Dixon image series (TE = 2.87 ms). To improve the reproducibility and robustness of radiomics features, the voxel intensity range was normalized and quantized to 128 gray levels (31–33).

Radiomics features were extracted using Python version 3.8 (www.python.org) and the *PyRadiomics* package version 3.0 (34). A total of 107 features were extracted for each ROI.

### Feature Selection and Model Building

Feature selection and dimension reduction methods were applied to prevent overfitting (35). Pearsons's correlation coefficient was used to test collinearity, and a heatmap was generated to demonstrate the collinearity between all extracted features. The least absolute shrinkage and selection operator (LASSO) regression and 10-fold cross-validation were employed to reduce the high dimension of all extracted features and select the most robust prognostic features among them as recommended (36). Bayesian information criterion was used for final feature selection. *The Image Biomarker Standardization Initiative (IBSI)* reference manual was used for feature definitions and calculations (37).

Logistic regression was used to build prediction models to predict the CP-affected muscle, i.e., spasticity. The radiomics model (RM), the fat fraction model (FFM), and a combination of both models (CM) were built for each one of the calf muscles separately. The receiver operating characteristic (ROC) curve and their respective area under the curve (AUC) values were used to evaluate model performance. A DeLong's test was used to compare performances of the three models (i.e., AUC values) within a muscle. A goodness-of-fit test was employed to assess how well the models were fitted. To compare the agreement between the actual and the predicted outcome, calibration curves were generated for the final models.

The feature selection and model building steps were performed in the 12 dominant legs of volunteers, 6 affected legs of patients with unilateral spastic hemiparesis, and the 3 more affected legs of bilaterally affected patients.

### Statistical Analysis

Statistical analysis was performed with Stata/IC 15.1 (StataCorp LP, College Station, Texas), and the *lassopack* (38) package was used. All continuous data, i.e., age, height, BMI, were given as either means and standard deviation or median and interquartile range. The group differences (CP vs. healthy) were assessed using Student's *t*-test or chi-squared test, where appropriate. Shapiro-Wilk test was used to assess the normality of the distributions. Alpha level was set to 0.05.

## Results

### Study Population and Fat Fraction

In total, 21 lower limbs (9 affected, 12 healthy) were included.

**Figure 2:**
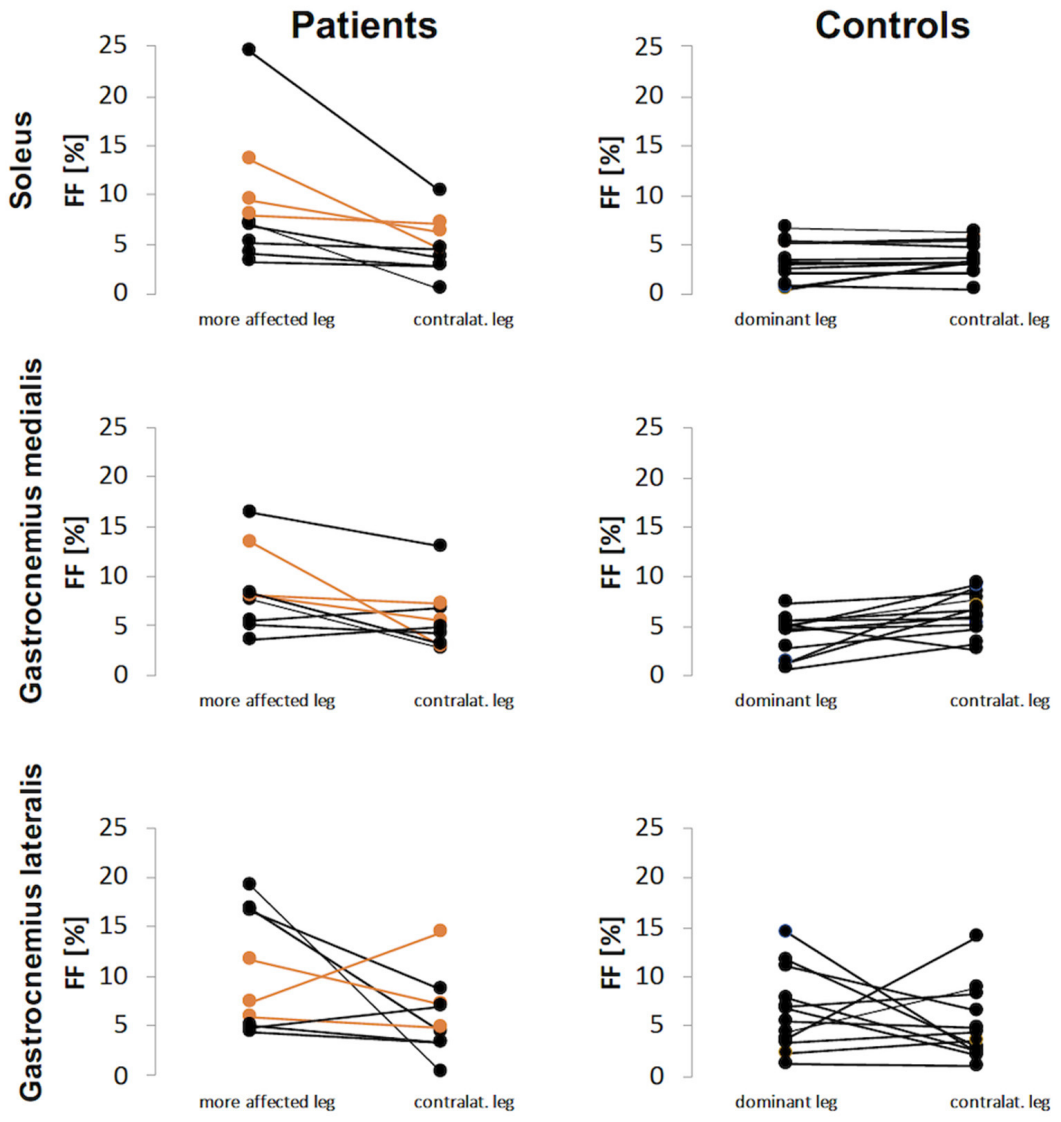
Fat fraction (FF) values for patients and controls for each calf muscle. The FF results of patients with diparesis are depicted in orange.

Mean FF values were higher in the affected legs of the patients with unilateral spastic hemiparesis and more affected legs of patients with spastic diparesis than dominant legs of the healthy controls. While CP-affected and more affected legs had FF_S_ 9.1%, FF_GM_ 8.5% and FF_GL_ 10.2%, the control legs had FF_S_ 3.3%, FF_GM_ 4.1%, FF_GL_ 6.6% (*p-*values 0.009, 0.005, 0.116, respectively). The FF difference between dominant and non-dominant legs in the control group was not apparent for S and GM muscles, whereas the difference was pronounced for GL. Similarly, the FF values in the contralateral leg of patients were lower than affected/more affected leg for S and GM muscles, but this difference was not evident for GL. An overview of the FF values is given in Figure 2.

### Feature Selection

**Figure 3:**
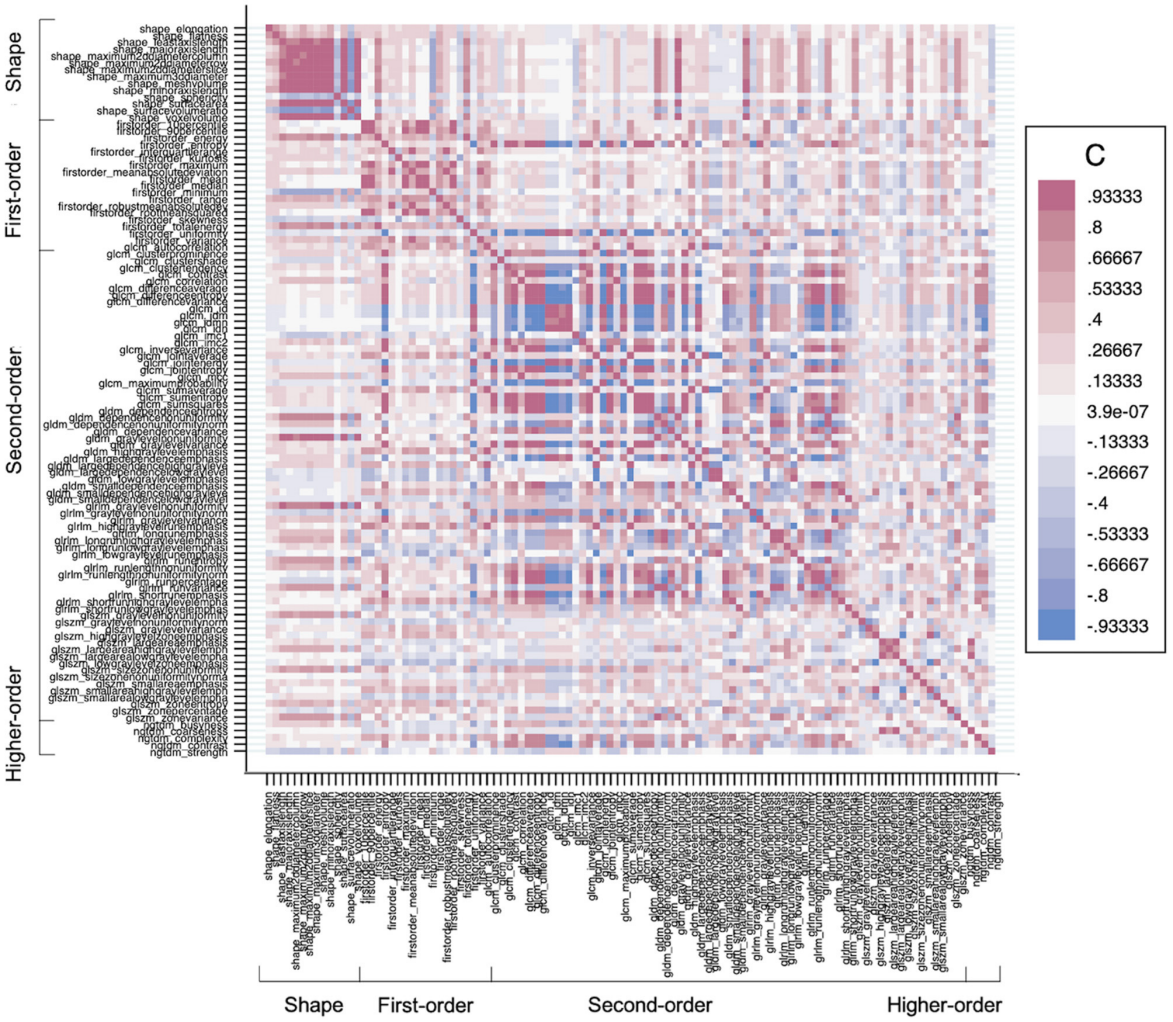
A heatmap demonstrates the collinearity between all extracted features—correlation coefficient (C) range between −1 and +1. Red depicts the perfect positive correlation, and blue depicts the perfect negative correlation, and all other colors depict correlation in between on the heat map. The higher the C in each direction, the more redundant the feature is.

Feature selection was performed for the entire dataset. All selected features and their values are reported in Supplementary Tables 1–3. The LASSO regression model successfully reduced the dimensionality of 107 features and selected the most robust ones (Supplementary Figure 1). Since LASSO also accounts for collinearity, no further steps were taken in the Pearson correlation coefficient. A correlation heatmap of all extracted features shown in Figure 3 and depicts little redundancy. All chosen texture features with a non-zero coefficient in the LASSO regression are reported in Table 3. A different set of features were selected for each calf muscle. The selected features belonged to 2D shape-based (maximum 2D diameter row, surface volume ratio), gray level co-occurrence matrix (information correlation 1, cluster shade), and gray level size zone matrix (small area low gray-level emphasis, small area emphasis) feature classes (Table 3 and Supplementary Data).

**Table 3:** All selected texture features and their values, LASSO coefficients, and IBSI reference values.

**Muscle**	**Selected Features**	**Feature Value**		**LASSO coefficient**	IBSI Reference value^*^	**Student's t-test *P* value**
		**Healthy (*n* = 12)**	**Cerebral palsy (*n* = 9)**			
Soleus	GLCM cluster shade	−0.1 ± 0.3	−0.1 ± 0.3	−0.17	7.0	0.560
	GLCM information correlation 1	−0.3 ± 0.1	−0.2 ± 0.1	0.12	−0.1	0.043
	GLSZM Small area low gray-level emphasis	0.4± 0.1	0.2± 0.2	−0.62	0.02	0.044
Gastrocnemius medialis	Shape maximum 2D diameter row	52.9 ± 7.5	40.1 ± 7.4	−0.01	13.1	<0.001
	GLSZM small area emphasis	0.6± 0.1	0.5± 0.1	−0.07	0.3	0.017
Gastrocnemius lateralis	Shape surface volume ratio	0.4 ± 0.1	0.5 ± 0.1	0.60	0.7	0.005

*Data are means ± standard deviations*.

*LASSO, least absolute shrinkage and selection operator; IBSI, Image Biomarker Standardization Initiative; GLCM, gray level co-occurrence matrix; GLSZM, gray level size zone matrix*.

* *Reference values that are reported in IBSI reference manual for digital phantom at the highest consensus level*.

### Predictive Models

**Figure 4:**
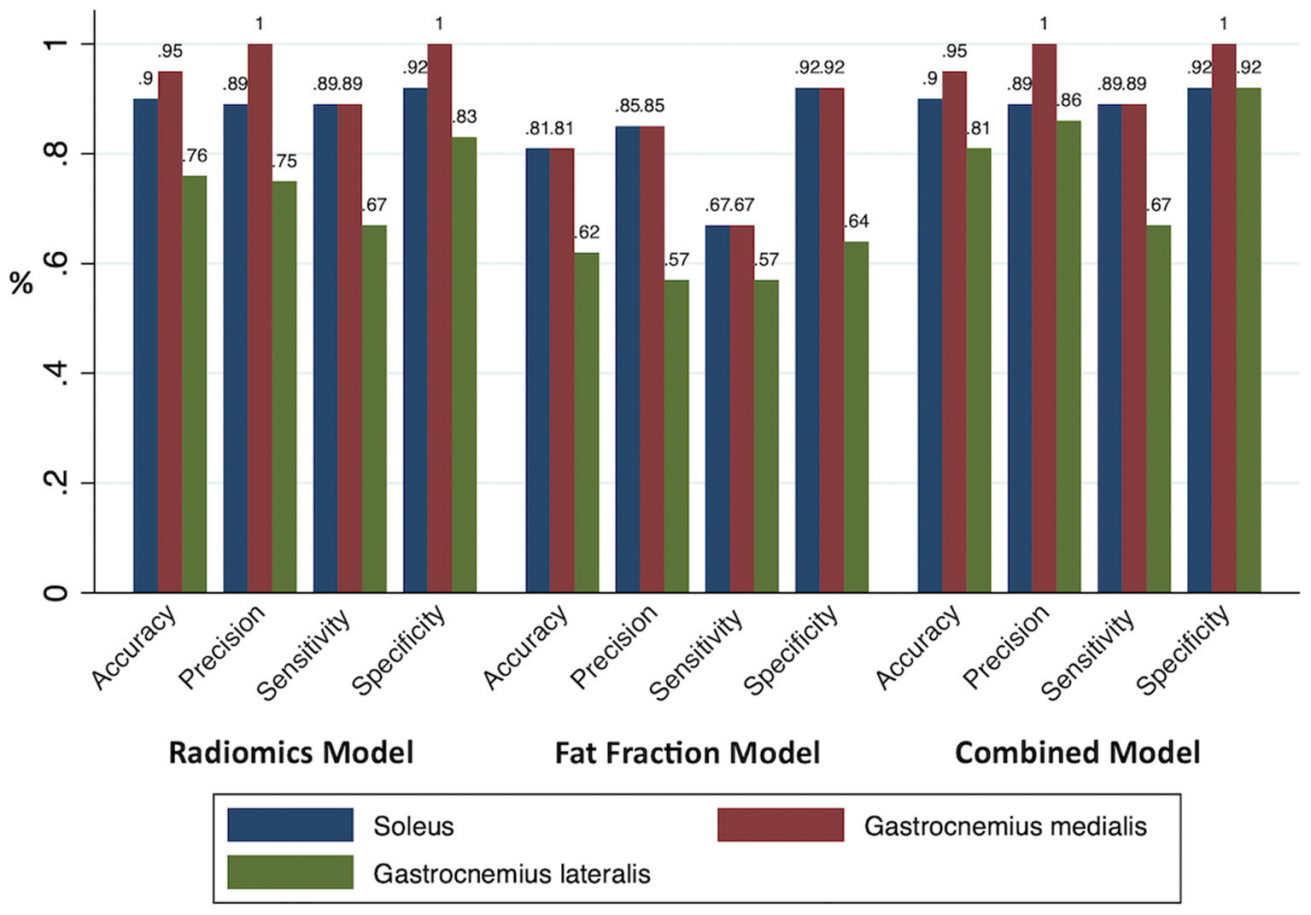
All model performances. The accuracy, precision, sensitivity, and specificity of the prediction models were based on the radiomics, fat fraction, and combined model built for each muscle.

**Figure 5:**
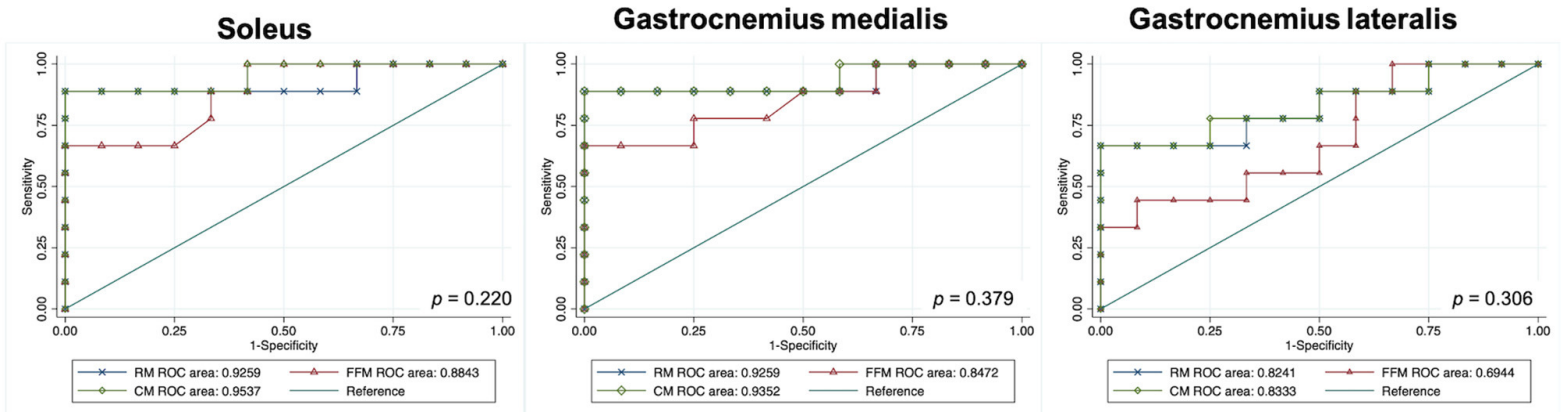
Graphs show receiver operating characteristic curves indicating the accuracy of models for predicting disease in children with cerebral palsy. DeLong's test *P* values. GM, gastrocnemius medialis; GL, gastrocnemius lateralis; RM, radiomics model; FFM, fat fraction model; CM, combined model; RM, radiomics model; FFM, fat fraction model; CM, combined model; ROC, receiver operating characteristic.

An RM, an FFM, and a CM were built for each muscle separately. All model performances are reported in Figure 4. Based on the ROC analysis, the performance of RM was excellent for soleus and gastrocnemius medialis and very good for gastrocnemius lateralis (AUC_S_ 0.92; AUC_GM_ 0.92, AUC_GL_ 0.82). The FFM always showed good performance for soleus and gastrocnemius medialis and moderate performance for gastrocnemius lateralis (AUC_S_ 0.88; AUC_GM_ 0.85; AUC_GL_ 0.69). The combination of both models always had a better performance than RM according to ROC analysis (AUC_S_ 0.95; AUC_GM_ 0.93; AUC_GL_ 0.83) (Figure 5). The sensitivity of RM was between 67 and 89%, and specificity was between 83 and 100%. The accuracy of RM was always higher than FFM, and the CM model accuracy was either better than or comparable to RM (Figure 4). The calibration curves of the final combination models showed a high level of agreement between the prediction of the affected muscle and actually affected muscle (Supplementary Figure 2).

## Discussion

In this study, we employed radiomics analysis of MR images in CP patients and healthy controls to discriminate affected muscles from healthy ones. We compared the performance of the RM with the FFM as well as with the combination of both. Our radiomics analysis yielded a better performing model than the FF analysis. Moreover, we found that the combination of both models always performed better.

So far, quantitative MRI techniques—in particular, FF analysis—have been employed to explore disease severity or to monitor treatment response in muscle dystrophies (5–8). Muscle FF analysis has been rarely used to assess the functional capacity of CP patients, and researchers have reported higher fat quantity in muscles of CP patients than in a healthy population (13, 14). In our analysis, the patient who had the highest FF results for all muscles was obese with a BMI of 95 percentile. On the other hand, the patient with the second highest FF results was underweight with BMI 3 percentile and had the most severe fixed contracture. All other patients were normal weighted and had lower FF values than aforementioned 2 patients. In line with previous studies, our FF results were higher in the patient group than the control group regardless of their BMI. The FF values in our patient group are similar to the values in hemiparetic children reported by D'Souza et al. (14), and lower than the values in biparetic young adults that reported by Noble et al. (13). Although FF could be a useful tool to assess muscle diseases, it is usually restricted to a single value estimation of each muscle, disregarding the inhomogeneity of muscle structure, especially in the presence of pathology.

A less expensive and easily accessible alternative to MRI is ultrasonography (US). The US can be employed to detect basic structural muscle changes and assess muscle volume in CP patients (39, 40). Nonetheless, it is highly user-dependent and does not provide a global view of all muscles (39). Although new emerging US techniques, e.g., sheer-wave elastography, can make functional predictions (40), the composition of muscle, in particular the fat fraction, cannot be detected using only US (39). On the other hand, MRI can be used not only for global assessment of muscles but also for compositional assessment.

Texture analysis of diagnostic images is a non-invasive tool that can shed light on the underlying pathophysiology. Radiomics analysis can guide biopsies and play a role in following up the disease progression by longitudinal radiomics analysis, so called delta radiomics (41). MRI texture analysis has demonstrated to be a potential tool to evaluate neuromuscular muscle disorders in animal models (21, 22, 25–27), and preliminary studies already established some texture biomarkers for assessing disease progression in a dog model of muscular dystrophies (25, 27). Moreover, recent studies revealed that MRI texture analysis could help investigate the effects of repetitive forces in healthy athletes by detecting texture changes due to muscle hypertrophy (23, 24). So far, only a few studies have applied texture analysis to various pathologic skeletal muscles, e.g., muscle dystrophy, in human subjects (28, 29). Researchers explored the correlations between texture analysis and the disease status and found muscle texture features helpful for objective evaluation of MRI (28, 29). To our knowledge, our study is the first one that applied radiomics analysis to the MRI of skeletal muscles of children with CP.

In our study, we applied an FF analysis along with texture analysis to predict CP affected muscles. The FF analysis has been performed based on multi-echo Dixon acquisition with 6 echoes, as at least 3 echoes (Dixon points) are recommended to overcome main field inhomogeneities (42). Images of the second echo (TE = 2.87 ms) were used for feature extraction. The RM always performed better than FFM in discriminating affected muscles with an accuracy between 76 and 95%. Furthermore, the combination of those methods showed an excellent performance level with an accuracy between 81 and 95%. Although fat quantification with FF has a relevant role in evaluating the muscles of the CP patients, our FFM resulted in only moderate/good performance level with accuracy between 62 and 81%. This might be due to the calf muscles' immediate proximity to subcutaneous fat tissue, which was especially prominent on GL FF analysis. Despite this issue being addressed by eroding the ROIs by one voxel, the performance of the FFM was lower than RM or CM. On the other hand, RM always reached a high-performance level. Therefore, combining radiomics and FF methods might especially be recommended for assessing muscles adjacent to subcutaneous fat tissue.

We employed the LASSO regression for dimension reduction and feature selection since those steps are the pillars of the texture analysis and, consequently, help avoid overfitting (35, 36). Higher-order statistics features were eliminated by LASSO, presumably due to their sensitivity to noise. In contrast, second-order statistics and the shape features are less affected by noise and, therefore, more robust (20, 37). Hence, our LASSO analysis mostly selected the shape and second-order features instead of higher-order statistics features. Shape features define the two-dimensional size and shape of the ROI (37). These features are independent of the gray level intensity distribution in the ROI (37). Shape features were the one of the most successful features in our analysis, and they could be employed as an imaging biomarker for CP patients since the normal shape of the muscle can be drastically altered due to spasticity and contractions (4). The co-occurrence matrix depicts the frequency of a pair of pixels with the same value in a specified spatial range within an ROI (37). Co-occurrence matrix features were also successful in our study since they can reveal the texture heterogeneity due to fat infiltration within muscle tissue in CP patients. Gray level distance zone-based features depict the frequency of groups (zones) of the same gray-level appear in every direction within a voxel (37). Other successful features were belonging to this group. Fat infiltration can change the gray levels, and since this feature takes into account the neighboring voxel relations, it can reveal the extension of the fat infiltration. The RM reached a high level of performance to discriminate CP-affected muscles from the normal ones. It is of particular interest that although the spasticity was reduced at the time of the imaging, the muscle alterations, which could be due to remaining contractions or subtle structural changes, were successfully detected in radiomics analysis.

Our study had some limitations. Firstly, our study population was small. However, acquiring MRI data from a specific cohort, i.e., children with spasticity who can comply with MRI examination without sedation, was particularly challenging. Secondly, a single observer performed all segmentations, and segmentations were done manually. Yet, another observer controlled the segmentations against errors. It is well-known that inter-rater agreement is low in segmentation tasks, and although automated segmentation methods are desirable, manual segmentation by a single reader still provides a high degree of reliability for the reproducibility of radiomics features (20). Our patient group had fixed calf contractures, and it is known that muscle changes in patients with fixed contractures are more dramatic than the patients with dynamic contractures (2). Therefore, studies in a heterogeneous patient group needed to demonstrate the applicability of our model on less pronounced contractures. Moreover, we did not have a radiology-pathology correlation; therefore, the true relation between radiomics or fat fraction analysis and muscle histopathology still needs to be elucidated. Nevertheless, radiomics analysis of skeletal muscles is a promising tool to provide non-invasive tissue characterization and reduce muscle biopsies since it is particularly important to avoid unnecessary interventions in the pediatric population, and muscle biopsies usually fail to capture tissue heterogeneity or to reflect the entire tissue. Still, radiology-pathology correlation studies are required to understand the relationship between muscle histopathology and imaging biomarkers. Lastly, we have not tested our model on an independent external dataset. Nevertheless, we did internal validation with 10-fold cross-validation; further studies are required for external validation of the proposed model.

In conclusion, the combination of MRI quantitative fat fraction analysis and texture analysis of muscles in CP patients is a promising tool to evaluate skeletal muscle involvement of the disease in a non-invasive manner. In the long term, our model could be integrated into clinical decision-making systems, and a similar approach might be used to assess other muscle diseases. Further investigations in a large cohort of patients with CP are needed to optimize and validate our proposed model in a clinical setting.

## Data Availability Statement

The datasets presented in this article are not readily available because restrictions apply to DICOM data originating from the MRI scanner. Requests to access the datasets should be directed to CW, claudia.weidensteiner@unibas.ch.

## Ethics Statement

The studies involving human participants were reviewed and approved by Ethikkommission Nordwest-und Zentralschweiz, Basel, Switzerland. Written informed consent to participate in this study was provided by the participants' legal guardian/next of kin.

## Author Contributions

TA, XD, FS, and CW: study concept, study design and data acquisition, analysis, and interpretation. RB and ER: clinical studies. TA, XD, FS, CW, and MG: diagnostic imaging. FS: segmentation tool. TA and XD: statistical analysis. TA, XD, and CW: literature search. TA: first draft of the manuscript. All authors: manuscript revision for important intellectual content and manuscript final version approval. All authors agree to ensure any questions related to the work are appropriately resolved.

## Conflict of Interest

The authors declare that the research was conducted in the absence of any commercial or financial relationships that could be construed as a potential conflict of interest.
