# Supplementary material for: Combination of Quantitative MRI Fat Fraction and Texture Analysis to Evaluate Spastic Muscles of Children With Cerebral Palsy
Source: Front Neurol. 2021 Mar 22;12:633808. doi: 10.3389/fneur.2021.633808 (PMC8019698; doi:10.3389/fneur.2021.633808)
Supplement: Supplementary file 1 [file Data_Sheet_1.DOCX]

Supplementary Material

Table of Contents

[1. Supplementary Data 1](#_Toc55996638)

[2. Supplementary Figures and Tables 3](#_Toc55996639)

[2.1. Supplementary Tables 3](#_Toc55996640)

[2.2. Supplementary Figures *12*](#_Toc55996641)

[3. References *13*](#_Toc55996642)

# Supplementary Data

- 1. **Definition of selected radiomics features**

Below we reported all selected features for model building in our study. For more details, please see the *Image Biomarker Standardization Initiative* (*IBSI*) reference manual and *PyRadiomics* documentation for all extracted feature definitions and their respective calculations [1,2].

- - 1. **Shape**

Morphological features describe geometric aspects of a region of interest (ROI), such as the area

and volume.

*Maximum 2D diameter row*

Maximum 2D diameter (row) is defined as the largest pairwise Euclidean distance between muscle surface mesh vertices in the column-slice (usually the sagittal) plane.

*Surface to volume ratio*

$$surface to volume ratio=\frac{A}{V}$$

- - 1. **Grey-level co-occurrence matrix**

The grey level co-occurrence matrix (GLCM) is a matrix that expresses how combinations of discretized intensities (grey levels) of neighboring pixels, or voxels in a 3D volume, are distributed along with one of the image directions.

- $P(i,j)$ is the co-occurrence matrix
- *N_g_* is the number of discrete grey levels in the image
- 𝜇 is the mean of $P(i,j)$,
- 𝜇_x_(𝑖) is the mean of 𝑃_𝑥_(𝑖) and 𝜇_y_(𝑗) is the mean of 𝑃_𝑦_(𝑗), *Px* and *Py* being the marginal probabilities
- σ_𝑥_(𝑖) is the standard deviation of 𝑃_𝑥_(𝑖), and σ_y_(𝑗) is the standard deviation of 𝑃_y_(𝑗),
- 𝑃_𝑥+y_(*k*) is $\sum_{i=1}^{N_{g}} \sum_{j=1}^{N_{g}} P\left( i,j \right)$ where *k* = *i* + *j*,
- 𝑃_𝑥-y_(*k*) is $\sum_{i=1}^{N_{g}} \sum_{j=1}^{N_{g}} P\left( i,j \right)$ where *k* = |*i* – *j|*,
- HX is the entropy of 𝑃_𝑥_(𝑖) and HY is the entropy of 𝑃_y_(*j*),
- HXY is the entropy of 𝑃(𝑖, *j*),
- HXY1 is $-\sum_{i=1}^{N_{g}} \sum_{j=1}^{N_{g}} P\left( i,j \right)\log_{2}(P_{x}\left( i \right)P_{y}\left( j \right)+\varepsilon)$,

1. *Information measure of correlation 1 (IMC1)*

$$IMC1=\frac{HXY-HXY1}{max\{HX,HY\}}$$

It assesses the correlation between the probability distributions of 𝑖i and 𝑗j (quantifying the complexity of the texture).

1. *Cluster Shade (CS)*[3]

$$CS=\sum_{i=1}^{N_{g}} \sum_{j=1}^{N_{g}} {(i+j-\mu_{i}\left( i \right)-\mu_{i}(j))}^{3}P\left( i,j \right)$$

It is a measure of the skewness and uniformity of the GLCM. A higher cluster shade implies greater asymmetry about the mean.

- - 1. **Gray-level size zone matrix**

The grey level size zone matrix (GLSZM) counts the number of groups (or zones) of linked voxels. Voxels are linked if the neighboring voxel has an identical discretized grey level.

- N𝑔be the number of discreet intensity values in the image
- *N𝑠* be the number of discreet zone sizes in the image
- 𝑁𝑝 be the number of voxels in the image
- 𝑁𝑧 be the number of zones in the ROI, which is equal to $\sum_{i=1}^{N_{g}} \sum_{j=1}^{N_{g}} P\left( i,j \right)$ and ≤1≤Nz≤Np
- **P***(i,j)* be the size zone matrix
- *p(i,j)* be the normalized size zone matrix, defined as $p(i,j)=\frac{\mathbf{P}\left( i,j \right)}{\boldsymbol{j}^{\boldsymbol{2}}}$

*Small area emphasis (SAE)*

#

$$\boldsymbol{SAE}\mathbf{=}\frac{\sum_{\boldsymbol{i=1}}^{\boldsymbol{N}_{\boldsymbol{g}}} \sum_{\boldsymbol{j=1}}^{\boldsymbol{N}_{\boldsymbol{s}}} \frac{\mathbf{P}\left( \boldsymbol{i,j} \right)}{\boldsymbol{j}^{\boldsymbol{2}}}}{\boldsymbol{N}_{\boldsymbol{z}}}$$

# Feature measures the distribution of small size zones, with a greater value indicative of more smaller size zones and more fine textures.

*Small area low gray level emphasis (SALGLE)*

$$SALGLE=\frac{\sum_{i=1}^{N_{g}} \sum_{j=1}^{N_{g}} \frac{\mathbf{P}\left( i,j \right)}{i^{2}j^{2}}}{N_{z}}$$

Feature measures the proportion in the image of the joint distribution of smaller size zones with lower gray-level values.

# 2. Supplementary Figures and Tables 2.1. Supplementary Tables

**2.1.1. Supplementary Table 1**. All features extracted from soleus muscle ROIs and feature values

| Feature | Healthy n=12 | Cerebral Palsy n=9 | *P* value |
| --- | --- | --- | --- |
| shape_flatness | 0.36 ± 0.03 | 0.34 ± 0.05 | 0.28 |
| shape_leastaxislength | 26.85 ± 5.24 | 21.45 ± 4.28 | 0.021 |
| shape_majoraxislength | 75.48 ± 13.26 | 64.38 ± 12.77 | 0.07 |
| shape_maximum2ddiametercolumn | 78.55 ± 10.55 | 72.02 ± 11.02 | 0.18 |
| shape_maximum2ddiameterrow | 66.34 ± 8.13 | 54.24 ± 8.62 | 0.004 |
| shape_maximum2ddiameterslice | 85.38 ± 14.94 | 69.51 ± 14.79 | 0.026 |
| shape_maximum3ddiameter | 92.04 ± 14.29 | 82.30 ± 13.61 | 0.13 |
| shape_meshvolume | 48220.58 ± 19158.14 | 28150.3± 13566.8 | 0.015 |
| shape_minoraxislength | 56.69 ± 7.89 | 47.33 ± 8.28 | 0.016 |
| shape_sphericity | 0.50 ± 0.02 | 0.50 ± 0.03 | 0.71 |
| shape_surfacearea | 12737.60 ± 3756.73 | 8958.49 ± 3094.88 | 0.024 |
| shape_surfacevolumeratio | 0.27 ± 0.03 | 0.33 ± 0.0 | 0.001 |
| shape_voxelvolume | 48296.25 ± 19165.32 | 28222.7 ± 13574.0 | 0.015 |
| firstorder_10percentile | 29.33 ± 5.02 | 33.33 ± 10.28 | 0.25 |
| firstorder_90percentile | 39.75 ± 7.15 | 44.33 ± 14.29 | 0.35 |
| firstorder_energy | 5.9e+07 ± 2.7e+07 | 5.0e+07 ± 3.3e+07 | 0.49 |
| firstorder_entropy | 0.41 ± 0.24 | 0.40 ± 0.42 | 0.99 |
| firstorder_interquartilerange |  |  | 0.46 |
| 2 | 0 (0.0%) | 1 (11.1%) | |
| 3 | 3 (25.0%) | 0 (0.0%) |  |
| 4 | 4 (33.3%) | 2 (22.2%) | |
| 5 | 2 (16.7%) | 2 (22.2%) | |
| 6 | 1 (8.3%) | 2 (22.2%) | |
| 7 | 1 (8.3%) | 1 (11.1%) | |
| 8 | 0 (0.0%) | 1 (11.1%) | |
| 9 | 1 (8.3%) | 0 (0.0%) |  |
| firstorder_kurtosis | 7.40 ± 2.83 | 7.61 ± 3.42 | 0.88 |
| firstorder_maximum | 50.50 ± 10.72 | 58.00 ± 18.08 | 0.25 |
| firstorder_meanabsolutedeviation | 3.26 ± 0.97 | 3.59 ± 1.49 | 0.54 |
| firstorder_mean | 34.69 ± 6.13 | 39.25 ± 12.53 | 0.28 |
| firstorder_median | 35.00 ± 6.37 | 39.78 ± 13.04 | 0.28 |
| firstorder_minimum | |  | 0.20 |
| 0 | 4 (33.3%) | 2 (22.2%) | |
| 1 | 3 (25.0%) | 1 (11.1%) | |
| 2 | 0 (0.0%) | 3 (33.3%) | |
| 3 | 2 (16.7%) | 0 (0.0%) |  |
| 4 | 1 (8.3%) | 0 (0.0%) |  |
| 5 | 2 (16.7%) | 1 (11.1%) | |
| 7 | 0 (0.0%) | 1 (11.1%) | |
| 19 | 0 (0.0%) | 1 (11.1%) | |
| firstorder_range | 48.58 ± 10.21 | 53.78 ± 18.11 | 0.41 |
| firstorder_robustmeanabsolutedev | 2.18 ± 0.71 | 2.37 ± 0.83 | 0.59 |
| firstorder_rootmeansquared | 34.98 ± 6.21 | 39.56 ± 12.67 | 0.29 |
| firstorder_skewness | -1.17 ± 0.74 | -1.20 ± 0.73 | 0.95 |
| firstorder_totalenergy | 5.9e+07 ± 2.7e+07 | 5.0e+07 ± 3.3e+07 | 0.49 |
| firstorder_uniformity | 0.85 ± 0.12 | 0.83 ± 0.20 | 0.81 |
| firstorder_variance | 21.64 ± 11.62 | 28.13 ± 21.32 | 0.38 |
| glcm_autocorrelation | 3.95 ± 0.45 | 4.67 ± 2.08 | 0.25 |
| glcm_clusterprominence | 0.52 ± 0.35 | 0.49 ± 0.66 | 0.91 |
| glcm_clustershade | -0.07 ± 0.30 | -0.15 ± 0.32 | 0.56 |
| glcm_clustertendency | 0.23 ± 0.21 | 0.25 ± 0.33 | 0.85 |
| glcm_contrast | 0.05 ± 0.04 | 0.08 ± 0.12 | 0.44 |
| glcm_correlation | 0.54 ± 0.17 | 0.45 ± 0.29 | 0.38 |
| glcm_differenceaverage | 0.05 ± 0.04 | 0.08 ± 0.12 | 0.45 |
| glcm_differenceentropy | 0.28 ± 0.14 | 0.32 ± 0.30 | 0.75 |
| glcm_differencevariance | 0.05 ± 0.03 | 0.06 ± 0.07 | 0.58 |
| glcm_id | 0.97 ± 0.02 | 0.96 ± 0.06 | 0.45 |
| glcm_idm | 0.97 ± 0.02 | 0.96 ± 0.06 | 0.45 |
| glcm_idmn | 0.99 ± 0.01 | 0.99 ± 0.01 | 0.42 |
| glcm_idn | 0.98 ± 0.01 | 0.98 ± 0.02 | 0.83 |
| glcm_imc1 | -0.32 ± 0.15 | -0.18 ± 0.15 | 0.043 |
| glcm_imc2 | 0.43 ± 0.18 | 0.29 ± 0.25 | 0.15 |
| glcm_inversevariance | 0.05 ± 0.04 | 0.08 ± 0.12 | 0.45 |
| glcm_jointaverage | 1.97 ± 0.12 | 2.09 ± 0.52 | 0.44 |
| glcm_jointenergy | 0.81 ± 0.15 | 0.78 ± 0.27 | 0.75 |
| glcm_jointentropy | 0.62 ± 0.39 | 0.66 ± 0.71 | 0.85 |
| glcm_mcc | 0.55 ± 0.17 | 0.48 ± 0.28 | 0.46 |
| glcm_maximumprobability | 0.89 ± 0.11 | 0.85 ± 0.21 | 0.56 |
| glcm_sumaverage | 3.95 ± 0.24 | 4.19 ± 1.04 | 0.44 |
| glcm_sumentropy | 0.56 ± 0.36 | 0.58 ± 0.60 | 0.94 |
| glcm_sumsquares | 0.07 ± 0.06 | 0.08 ± 0.11 | 0.73 |
| gldm_dependenceentropy | 3.17 ± 0.72 | 3.54 ± 1.05 | 0.36 |
| gldm_dependencenonuniformity | 16451.84 ± 10349.07 | 7714.92 ± 7136.31 | 0.043 |
| gldm_dependencenonuniformitynor | 0.32 ± 0.10 | 0.25 ± 0.12 | 0.14 |
| gldm_dependencevariance | 28.90 ± 6.06 | 29.74 ± 8.62 | 0.79 |
| gldm_graylevelnonuniformity | 41876.73 ± 19715.77 | 23809.3 ± 14730.6 | 0.033 |
| gldm_graylevelvariance | 0.08 ± 0.06 | 0.09 ± 0.11 | 0.73 |
| gldm_highgraylevelemphasis | 3.95 ± 0.44 | 4.68 ± 2.07 | 0.25 |
| gldm_largedependenceemphasis | 578.12 ± 41.33 | 534.64 ± 105.36 | 0.21 |
| gldm_largedependencehighgrayleve | 2303.62 ± 230.02 | 2436.41 ± 1073.57 | 0.68 |
| gldm_largedependencelowgraylevel | 157.68 ± 28.64 | 170.13 ± 165.78 | 0.80 |
| gldm_lowgraylevelemphasis | 0.29 ± 0.07 | 0.31 ± 0.26 | 0.81 |
| gldm_smalldependenceemphasis | 0.00 ± 0.00 | 0.01 ± 0.00 | 0.20 |
| gldm_smalldependencehighgrayleve | 0.02 ± 0.01 | 0.03 ± 0.02 | 0.10 |
| gldm_smalldependencelowgraylevel | 0.00 ± 0.00 | 0.00 ± 0.00 | 0.59 |
| glrlm_graylevelnonuniformity | 3870.99 ± 1107.69 | 3030.85 ± 1260.63 | 0.12 |
| glrlm_graylevelnonuniformitynorm | 0.62 ± 0.08 | 0.70 ± 0.19 | 0.21 |
| glrlm_graylevelvariance | 0.20 ± 0.05 | 0.17 ± 0.11 | 0.36 |
| glrlm_highgraylevelrunemphasis | 3.72 ± 0.80 | 4.43 ± 1.77 | 0.23 |
| glrlm_longrunemphasis | 138.70 ± 60.71 | 102.21 ± 61.35 | 0.19 |
| glrlm_longrunhighgraylevelemphas | 552.15 ± 241.87 | 413.00 ± 235.50 | 0.20 |
| glrlm_longrunlowgraylevelemphasi | 36.36 ± 14.88 | 35.75 ± 41.72 | 0.96 |
| glrlm_lowgraylevelrunemphasis | 0.39 ± 0.10 | 0.35 ± 0.25 | 0.63 |
| glrlm_runentropy | 4.61 ± 0.20 | 4.26 ± 0.41 | 0.017 |
| glrlm_runlengthnonuniformity | 662.23 ± 315.50 | 784.62 ± 1058.45 | 0.71 |
| glrlm_runlengthnonuniformitynorm | 0.10 ± 0.03 | 0.12 ± 0.08 | 0.38 |
| glrlm_runpercentage | 0.14 ± 0.04 | 0.18 ± 0.11 | 0.22 |
| glrlm_runvariance | 65.93 ± 24.82 | 42.91 ± 26.69 | 0.06 |
| glrlm_shortrunemphasis | 0.28 ± 0.07 | 0.29 ± 0.14 | 0.93 |
| glrlm_shortrunhighgraylevelempha | 0.92 ± 0.56 | 1.35 ± 1.11 | 0.25 |
| glrlm_shortrunlowgraylevelemphas | 0.16 ± 0.06 | 0.11 ± 0.03 | 0.021 |
| glszm_graylevelnonuniformity | 67.64 ± 35.51 | 40.26 ± 18.76 | 0.050 |
| glszm_graylevelnonuniformitynorm | 0.69 ± 0.22 | 0.61 ± 0.23 | 0.43 |
| glszm_graylevelvariance | 0.37 ± 0.36 | 0.51 ± 0.29 | 0.34 |
| glszm_highgraylevelzoneemphasis | 2.94 ± 2.22 | 3.99 ± 2.24 | 0.30 |
| glszm_largeareaemphasis | 2.5e+07 ± 2.1e+07 | 5.4e+07 ± 1.3e+08 | 0.44 |
| glszm_largeareahighgraylevelemph | 9.9e+07 ± 8.4e+07 | 9.0e+07 ± 1.2e+08 | 0.84 |
| glszm_largearealowgraylevelempha | 6.3e+06 ± 5.2e+06 | 4.6e+07 ± 1.3e+08 | 0.30 |
| glszm_lowgraylevelzoneemphasis | 0.74 ± 0.26 | 0.60 ± 0.28 | 0.26 |
| glszm_sizezonenonuniformity | 26.85 ± 8.24 | 21.91 ± 11.98 | 0.28 |
| glszm_sizezonenonuniformitynorma | 0.29 ± 0.05 | 0.36 ± 0.24 | 0.31 |
| glszm_smallareaemphasis | 0.55 ± 0.05 | 0.48 ± 0.19 | 0.22 |
| glszm_smallareahighgraylevelemph | 1.72 ± 1.36 | 2.31 ± 1.39 | 0.34 |
| glszm_smallarealowgraylevelempha | 0.39 ± 0.15 | 0.25 ± 0.16 | 0.044 |
| glszm_zoneentropy | 3.27 ± 0.38 | 3.07 ± 1.23 | 0.61 |
| glszm_zonepercentage | 0.00 ± 0.00 | 0.00 ± 0.00 | 0.11 |
| glszm_zonevariance | 2.4e+07 ± 2.1e+07 | 1.0e+07 ± 1.3e+07 | 0.09 |
| ngtdm_busyness | 315.07 ± 323.01 | 107.36 ± 139.27 | 0.09 |
| ngtdm_coarseness | 0.00 ± 0.00 | 1.1e+05 ± 3.3e+05 | 0.26 |
| ngtdm_complexity | 0.12 ± 0.13 | 0.48 ± 0.82 | 0.14 |
| ngtdm_contrast | 0.00 ± 0.01 | 0.00 ± 0.01 | 0.72 |
| ngtdm_strength | 0.00 ± 0.00 | 0.00 ± 0.00 | 0.011 |

Note.—Data are means ± standard deviations. GLCM = grey level co-occurrence matrix. GLDM = Gray Level Dependence Matrix. GLSZM = Gray Level Size Zone. GLRLM = Gray Level Run Length Matrix. NGTDM = Neighboring Gray Tone Difference Matrix.

**2.1.2. Supplementary Table 2.** All features extracted from gastrocnemius medialis muscle ROIs and feature values.

| Feature | Healthy n=12 | Cerebral Palsy n=9 | *P* value |
| --- | --- | --- | --- |
| shape_flatness | 0.32 ± 0.05 | 0.31 ± 0.05 | 0.44 |
| shape_leastaxislength | 19.82 ± 2.84 | 15.31 ± 2.90 | 0.002 |
| shape_majoraxislength | 61.83 ± 10.19 | 50.21 ± 8.85 | 0.013 |
| shape_maximum2ddiametercolumn | 65.71 ± 10.73 | 54.56 ± 10.42 | 0.028 |
| shape_maximum2ddiameterrow | 52.87 ± 7.47 | 40.07 ± 7.43 | < 0.001 |
| shape_maximum2ddiameterslice | 65.99 ± 13.39 | 51.66 ± 9.92 | 0.014 |
| shape_maximum3ddiameter | 70.83 ± 12.50 | 58.29 ± 10.89 | 0.027 |
| shape_meshvolume | 30597.21 ± 13784.62 | 16168.5 ± 9216.9 | 0.014 |
| shape_minoraxislength | 43.13 ± 7.12 | 34.05 ± 7.55 | 0.011 |
| shape_sphericity | 0.58 ± 0.02 | 0.60 ± 0.04 | 0.18 |
| shape_surfacearea | 7980.24 ± 2310.97 | 5056.5 ± 1884.36 | 0.006 |
| shape_surfacevolumeratio | 0.28 ± 0.05 | 0.34 ± 0.06 | 0.014 |
| shape_voxelvolume | 30660.42 ± 13792.80 | 16215.7 ± 9223.6 | 0.014 |
| firstorder_10percentile | 29.75 ± 5.55 | 33.44 ± 11.05 | 0.33 |
| firstorder_90percentile | 36.75 ± 6.82 | 40.78 ± 11.46 | 0.33 |
| firstorder_energy | 3.2e+07 ± 1.1e+07 | 2.5e+0 ± 1.7e+07 | 0.26 |
| firstorder_entropy | 0.33 ± 0.32 | 0.32 ± 0.31 | 0.96 |
| firstorder_interquartilerange |  |  | 0.26 |
| 2 | 3 (25.0%) | 1 (11.1%) | |
| 3 | 4 (33.3%) | 6 (66.7%) | |
| 4 | 2 (16.7%) | 2 (22.2%) | |
| 5 | 3 (25.0%) | 0 (0.0%) |  |
| firstorder_kurtosis | 7.74 ± 3.46 | 8.91 ± 2.65 | 0.41 |
| firstorder_maximum | 50.00 ± 10.25 | 53.33 ± 12.92 | 0.52 |
| firstorder_meanabsolutedeviation | 2.23 ± 0.53 | 2.42 ± 0.36 | 0.36 |
| firstorder_mean | 33.10 ± 6.24 | 37.03 ± 11.53 | 0.33 |
| firstorder_median | |  | 0.54 |
| 13 | 0 (0.0%) | 1 (11.1%) | |
| 25 | 1 (8.3%) | 0 (0.0%) |  |
| 26 | 2 (16.7%) | 0 (0.0%) |  |
| 28 | 1 (8.3%) | 0 (0.0%) |  |
| 29 | 0 (0.0%) | 1 (11.1%) | |
| 31 | 1 (8.3%) | 0 (0.0%) |  |
| 32 | 1 (8.3%) | 1 (11.1%) | |
| 33 | 1 (8.3%) | 0 (0.0%) |  |
| 36 | 0 (0.0%) | 1 (11.1%) | |
| 38 | 3 (25.0%) | 1 (11.1%) | |
| 39 | 1 (8.3%) | 1 (11.1%) | |
| 45 | 1 (8.3%) | 1 (11.1%) | |
| 51 | 0 (0.0%) | 1 (11.1%) | |
| 52 | 0 (0.0%) | 1 (11.1%) | |
| firstorder_minimum | |  | 0.58 |
| 1 | 1 (8.3%) | 0 (0.0%) |  |
| 3 | 2 (16.7%) | 1 (11.1%) | |
| 5 | 1 (8.3%) | 2 (22.2%) | |
| 7 | 0 (0.0%) | 1 (11.1%) | |
| 8 | 1 (8.3%) | 1 (11.1%) | |
| 9 | 1 (8.3%) | 0 (0.0%) |  |
| 10 | 2 (16.7%) | 0 (0.0%) |  |
| 11 | 1 (8.3%) | 0 (0.0%) |  |
| 13 | 1 (8.3%) | 1 (11.1%) | |
| 15 | 1 (8.3%) | 0 (0.0%) |  |
| 18 | 0 (0.0%) | 2 (22.2%) | |
| 20 | 1 (8.3%) | 1 (11.1%) | |
| firstorder_range | 41.00 ± 9.96 | 42.56 ± 10.57 | 0.73 |
| firstorder_robustmeanabsolutedev | 1.59 ± 0.37 | 1.53 ± 0.20 | 0.66 |
| firstorder_rootmeansquared | 33.24 ± 6.26 | 37.22 ± 11.46 | 0.32 |
| firstorder_skewness | -0.85 ± 0.57 | -1.05 ± 1.21 | 0.62 |
| firstorder_totalenergy | 3.2e+07 ± 1.1e+07 | 2.5e+0 ± 1.7e+07 | 0.26 |
| firstorder_uniformity | 0.87 ± 0.16 | 0.87 ± 0.15 | 0.93 |
| firstorder_variance | 9.58 ± 4.05 | 12.79 ± 4.66 | 0.11 |
| glcm_autocorrelation | 3.80 ± 0.41 | 4.50 ± 2.10 | 0.27 |
| glcm_clusterprominence | 0.34 ± 0.38 | 0.29 ± 0.32 | 0.77 |
| glcm_clustershade | -0.12 ± 0.24 | -0.11 ± 0.22 | 0.93 |
| glcm_clustertendency | 0.19 ± 0.26 | 0.16 ± 0.21 | 0.76 |
| glcm_contrast | 0.05 ± 0.05 | 0.06 ± 0.07 | 0.63 |
| glcm_correlation | 0.40 ± 0.18 | 0.35 ± 0.21 | 0.55 |
| glcm_differenceaverage | 0.05 ± 0.05 | 0.06 ± 0.07 | 0.64 |
| glcm_differenceentropy | 0.25 ± 0.21 | 0.28 ± 0.26 | 0.76 |
| glcm_differencevariance | 0.04 ± 0.04 | 0.05 ± 0.06 | 0.69 |
| glcm_id | 0.98 ± 0.03 | 0.97 ± 0.04 | 0.64 |
| glcm_idm | 0.98 ± 0.03 | 0.97 ± 0.04 | 0.64 |
| glcm_idmn | 0.99 ± 0.01 | 0.99 ± 0.01 | 0.70 |
| glcm_idn | 0.98 ± 0.02 | 0.98 ± 0.02 | 0.92 |
| glcm_imc1 | -0.22 ± 0.10 | -0.20 ± 0.17 | 0.66 |
| glcm_imc2 | 0.31 ± 0.23 | 0.26 ± 0.18 | 0.59 |
| glcm_inversevariance | 0.05 ± 0.05 | 0.06 ± 0.07 | 0.65 |
| glcm_jointaverage | 1.94 ± 0.12 | 2.06 ± 0.52 | 0.44 |
| glcm_jointenergy | 0.83 ± 0.20 | 0.84 ± 0.20 | 0.98 |
| glcm_jointentropy | 0.51 ± 0.52 | 0.51 ± 0.53 | 0.98 |
| glcm_mcc | 0.43 ± 0.16 | 0.38 ± 0.19 | 0.51 |
| glcm_maximumprobability | 0.90 ± 0.14 | 0.90 ± 0.13 | 0.93 |
| glcm_sumaverage | 3.87 ± 0.25 | 4.12 ± 1.03 | 0.44 |
| glcm_sumentropy | 0.46 ± 0.46 | 0.45 ± 0.46 | 0.96 |
| glcm_sumsquares | 0.06 ± 0.08 | 0.06 ± 0.07 | 0.88 |
| gldm_dependenceentropy | 3.07 ± 0.83 | 3.34 ± 0.90 | 0.48 |
| gldm_dependencenonuniformity | 9781.28 ± 5260.56 | 4956.9± 5626.46 | 0.06 |
| gldm_dependencenonuniformitynorm | 0.32 ± 0.11 | 0.26 ± 0.14 | 0.24 |
| gldm_dependencevariance | 28.14 ± 8.83 | 31.30 ± 10.87 | 0.47 |
| gldm_graylevelnonuniformity | 25779.41 ± 10672.87 | 14445.6 ± 9768.7 | 0.022 |
| gldm_graylevelvariance | 0.07 ± 0.08 | 0.06 ± 0.07 | 0.94 |
| gldm_highgraylevelemphasis | 3.80 ± 0.41 | 4.49 ± 2.08 | 0.27 |
| gldm_largedependenceemphasis | 580.17 ± 45.85 | 549.07 ± 64.62 | 0.21 |
| gldm_largedependencehighgrayleve | 2253.28 ± 312.62 | 2445.87 ± 955.25 | 0.52 |
| gldm_largedependencelowgraylevel | 164.05 ± 34.04 | 172.74 ± 152.45 | 0.85 |
| gldm_lowgraylevelemphasis | 0.31 ± 0.09 | 0.32 ± 0.25 | 0.90 |
| gldm_smalldependenceemphasis | 0.00 ± 0.00 | 0.01 ± 0.00 | 0.37 |
| gldm_smalldependencehighgrayleve | 0.01 ± 0.01 | 0.02 ± 0.02 | 0.20 |
| gldm_smalldependencelowgraylevel | 0.00 ± 0.00 | 0.00 ± 0.00 | 0.61 |
| glrlm_graylevelnonuniformity | 2687.60 ± 1110.59 | 1659.62 ± 626.76 | 0.022 |
| glrlm_graylevelnonuniformitynorm | 0.70 ± 0.16 | 0.72 ± 0.18 | 0.87 |
| glrlm_graylevelvariance | 0.15 ± 0.08 | 0.14 ± 0.09 | 0.91 |
| glrlm_highgraylevelrunemphasis | 3.56 ± 0.79 | 4.17 ± 1.66 | 0.28 |
| glrlm_longrunemphasis | 140.33 ± 64.06 | 107.38 ± 79.52 | 0.31 |
| glrlm_longrunhighgraylevelemphas | 553.78 ± 265.21 | 431.86 ± 313.55 | 0.35 |
| glrlm_longrunlowgraylevelemphasi | 37.18 ± 14.31 | 33.87 ± 30.82 | 0.75 |
| glrlm_lowgraylevelrunemphasis | 0.39 ± 0.15 | 0.36 ± 0.23 | 0.71 |
| glrlm_runentropy | 4.46 ± 0.22 | 4.13 ± 0.32 | 0.012 |
| glrlm_runlengthnonuniformity | 545.67 ± 580.82 | 330.45 ± 293.11 | 0.32 |
| glrlm_runlengthnonuniformitynorm | 0.10 ± 0.05 | 0.12 ± 0.06 | 0.49 |
| glrlm_runpercentage | 0.14 ± 0.05 | 0.17 ± 0.06 | 0.23 |
| glrlm_runvariance | 57.98 ± 20.79 | 40.37 ± 25.48 | 0.10 |
| glrlm_shortrunemphasis | 0.27 ± 0.11 | 0.27 ± 0.13 | 0.88 |
| glrlm_shortrunhighgraylevelempha | 0.78 ± 0.42 | 1.13 ± 0.97 | 0.27 |
| glrlm_shortrunlowgraylevelemphas | 0.16 ± 0.11 | 0.12 ± 0.07 | 0.30 |
| glszm_graylevelnonuniformity | 42.41 ± 31.23 | 30.17 ± 23.25 | 0.34 |
| glszm_graylevelnonuniformitynorm | 0.68 ± 0.18 | 0.71 ± 0.19 | 0.67 |
| glszm_graylevelvariance | 0.39 ± 0.37 | 0.27 ± 0.26 | 0.40 |
| glszm_highgraylevelzoneemphasis | 3.03 ± 2.21 | 3.33 ± 2.42 | 0.77 |
| glszm_largeareaemphasis | 1.5e+07 ± 8.2e+06 | 1.2e+0 ± 1.3e+07 | 0.52 |
| glszm_largeareahighgraylevelemph | 5.8e+07 ± 3.4e+07 | 4.0e+0± 4.2e+07 | 0.27 |
| glszm_largearealowgraylevelempha | 4.0e+0± 2.0e+0 | 5.5e+0 ± 1.0e+07 | 0.61 |
| glszm_lowgraylevelzoneemphasis | 0.73 ± 0.24 | 0.63 ± 0.32 | 0.46 |
| glszm_sizezonenonuniformity | 22.58 ± 19.49 | 10.76 ± 7.87 | 0.10 |
| glszm_sizezonenonuniformitynorma | 0.34 ± 0.08 | 0.27 ± 0.07 | 0.043 |
| glszm_smallareaemphasis | 0.59 ± 0.08 | 0.49 ± 0.10 | 0.017 |
| glszm_smallareahighgraylevelemph | 1.81 ± 1.38 | 1.72 ± 1.47 | 0.89 |
| glszm_smallarealowgraylevelempha | 0.43 ± 0.17 | 0.30 ± 0.14 | 0.08 |
| glszm_zoneentropy | 2.81 ± 0.40 | 2.84 ± 0.39 | 0.88 |
| glszm_zonepercentage | 0.00 ± 0.00 | 0.00 ± 0.00 | 0.26 |
| glszm_zonevariance | 1.5e+07 ± 8.1e+06 | 1.1e+07 ± 1.1e+0 | 0.37 |
| ngtdm_busyness | 361.55 ± 576.64 | 66.31 ± 58.55 | 0.14 |
| ngtdm_coarseness | 0.00 ± 0.00 | 0.01 ± 0.03 | 0.20 |
| ngtdm_complexity | 0.08 ± 0.07 | 0.21 ± 0.31 | 0.16 |
| ngtdm_contrast | 0.01 ± 0.01 | 0.00 ± 0.01 | 0.41 |
| ngtdm_strength | 0.01 ± 0.01 | 0.02 ± 0.05 | 0.25 |

Note.—Data are means ± standard deviations. GLCM = grey level co-occurrence matrix. GLDM = Gray Level Dependence Matrix. GLSZM = Gray Level Size Zone. GLRLM = Gray Level Run Length Matrix. NGTDM = Neighboring Gray Tone Difference Matrix.

**2.1.3. Supplementary Table 3.** All features extracted from gastrocnemius lateralis muscle ROIs and feature values.

| Feature | Healthy n=12 | Cerebral Palsy n=9 | *P* value |
| --- | --- | --- | --- |
| shape_flatness | 0.28 ± 0.03 | 0.30 ± 0.05 | 0.32 |
| shape_leastaxislength | 14.32 ± 3.14 | 11.76 ± 1.74 | 0.041 |
| shape_majoraxislength | 51.49 ± 10.19 | 40.32 ± 7.32 | 0.012 |
| shape_maximum2ddiametercolumn | 56.42 ± 13.80 | 42.92 ± 8.60 | 0.019 |
| shape_maximum2ddiameterrow | 44.75 ± 5.00 | 36.71 ± 10.14 | 0.027 |
| shape_maximum2ddiameterslice | 51.38 ± 9.64 | 41.79 ± 8.58 | 0.029 |
| shape_maximum3ddiameter | 61.23 ± 13.11 | 46.47 ± 7.98 | 0.008 |
| shape_meshvolume | 15513.23 ± 8392.72 | 7601.2 ± 5239.0 | 0.023 |
| shape_minoraxislength | 37.01 ± 5.07 | 28.44 ± 8.52 | 0.010 |
| shape_sphericity | 0.53 ± 0.02 | 0.54 ± 0.03 | 0.28 |
| shape_surfacearea | 5539.09 ± 2014.17 | 3332.5 ± 1443.6 | 0.012 |
| shape_surfacevolumeratio | 0.38 ± 0.06 | 0.48 ± 0.08 | 0.005 |
| shape_voxelvolume | 15570.25 ± 8401.43 | 7649.7 ± 5243.0 | 0.023 |
| firstorder_10percentile | 30.50 ± 5.85 | 34.33 ± 11.83 | 0.34 |
| firstorder_90percentile | 38.83 ± 7.31 | 42.22 ± 13.24 | 0.46 |
| firstorder_energy | 1.8e+07 ± 7.5e+06 | 1.4e+0 ± 1.2e+0 | 0.31 |
| firstorder_entropy | 0.29 ± 0.27 | 0.30 ± 0.28 | 0.89 |
| firstorder_interquartilerange | |  | 0.95 |
| 2 | 1 (8.3%) | 1 (11.1%) | |
| 3 | 4 (33.3%) | 4 (44.4%) | |
| 4 | 1 (8.3%) | 1 (11.1%) | |
| 5 | 3 (25.0%) | 2 (22.2%) | |
| 6 | 2 (16.7%) | 1 (11.1%) | |
| 7 | 1 (8.3%) | 0 (0.0%) |  |
| firstorder_kurtosis | 4.65 ± 1.56 | 6.33 ± 2.14 | 0.05 |
| firstorder_maximum | 48.42 ± 8.55 | 51.89 ± 14.60 | 0.50 |
| firstorder_meanabsolutedeviation | 2.67 ± 0.77 | 2.56 ± 0.60 | 0.73 |
| firstorder_mean | 34.79 ± 6.37 | 38.43 ± 12.61 | 0.40 |
| firstorder_median | 34.92 ± 6.29 | 38.67 ± 12.82 | 0.39 |
| firstorder_minimum | |  | 0.49 |
| 7 | 0 (0.0%) | 1 (11.1%) | |
| 8 | 2 (16.7%) | 0 (0.0%) |  |
| 9 | 1 (8.3%) | 0 (0.0%) |  |
| 11 | 1 (8.3%) | 2 (22.2%) | |
| 14 | 2 (16.7%) | 0 (0.0%) |  |
| 15 | 0 (0.0%) | 1 (11.1%) | |
| 17 | 1 (8.3%) | 1 (11.1%) | |
| 18 | 1 (8.3%) | 1 (11.1%) | |
| 19 | 1 (8.3%) | 0 (0.0%) |  |
| 20 | 1 (8.3%) | 0 (0.0%) |  |
| 21 | 2 (16.7%) | 1 (11.1%) | |
| 31 | 0 (0.0%) | 1 (11.1%) | |
| 33 | 0 (0.0%) | 1 (11.1%) | |
| firstorder_range | 33.42 ± 7.49 | 33.67 ± 9.49 | 0.95 |
| firstorder_robustmeanabsolutedev | 1.95 ± 0.54 | 1.73 ± 0.56 | 0.39 |
| firstorder_rootmeansquared | 34.97 ± 6.41 | 38.60 ± 12.59 | 0.40 |
| firstorder_skewness | -0.57 ± 0.31 | -0.62 ± 1.02 | 0.86 |
| firstorder_totalenergy | 1.8e+07 ± 7.5e+06 | 1.4e+0 ± 1.2e+0 | 0.31 |
| firstorder_uniformity | 0.89 ± 0.13 | 0.88 ± 0.13 | 0.89 |
| firstorder_variance | 12.91 ± 7.18 | 12.61 ± 4.50 | 0.91 |
| glcm_autocorrelation | 3.93 ± 0.38 | 3.67 ± 1.08 | 0.44 |
| glcm_clusterprominence | 0.30 ± 0.36 | 0.25 ± 0.25 | 0.71 |
| glcm_clustershade | -0.05 ± 0.26 | -0.06 ± 0.20 | 0.96 |
| glcm_clustertendency | 0.16 ± 0.22 | 0.14 ± 0.16 | 0.81 |
| glcm_contrast | 0.04 ± 0.05 | 0.07 ± 0.08 | 0.39 |
| glcm_correlation | 0.36 ± 0.20 | 0.26 ± 0.10 | 0.20 |
| glcm_differenceaverage | 0.04 ± 0.05 | 0.07 ± 0.08 | 0.39 |
| glcm_differenceentropy | 0.23 ± 0.19 | 0.30 ± 0.27 | 0.49 |
| glcm_differencevariance | 0.04 ± 0.04 | 0.06 ± 0.06 | 0.43 |
| glcm_id | 0.98 ± 0.02 | 0.97 ± 0.04 | 0.39 |
| glcm_idm | 0.98 ± 0.02 | 0.97 ± 0.04 | 0.39 |
| glcm_idmn | 0.99 ± 0.01 | 0.99 ± 0.01 | 0.45 |
| glcm_idn | 0.99 ± 0.02 | 0.98 ± 0.02 | 0.40 |
| glcm_imc1 | -0.20 ± 0.13 | -0.13 ± 0.05 | 0.11 |
| glcm_imc2 | 0.27 ± 0.22 | 0.20 ± 0.12 | 0.37 |
| glcm_inversevariance | 0.04 ± 0.05 | 0.07 ± 0.08 | 0.39 |
| glcm_jointaverage | 1.97 ± 0.10 | 1.88 ± 0.34 | 0.40 |
| glcm_jointenergy | 0.86 ± 0.16 | 0.84 ± 0.18 | 0.80 |
| glcm_jointentropy | 0.45 ± 0.44 | 0.50 ± 0.50 | 0.78 |
| glcm_mcc | 0.38 ± 0.20 | 0.27 ± 0.10 | 0.16 |
| glcm_maximumprobability | 0.92 ± 0.11 | 0.91 ± 0.12 | 0.81 |
| glcm_sumaverage | 3.94 ± 0.21 | 3.77 ± 0.69 | 0.40 |
| glcm_sumentropy | 0.40 ± 0.39 | 0.43 ± 0.42 | 0.86 |
| glcm_sumsquares | 0.05 ± 0.06 | 0.05 ± 0.06 | 0.97 |
| gldm_dependenceentropy | 3.37 ± 0.63 | 3.77 ± 0.71 | 0.19 |
| gldm_dependencenonuniformity | 4047.53 ± 2840.34 | 1606.4 ± 2077.8 | 0.043 |
| gldm_dependencenonuniformitynorm | 0.25 ± 0.08 | 0.17 ± 0.09 | 0.049 |
| gldm_dependencevariance | 31.09 ± 8.13 | 35.86 ± 9.43 | 0.23 |
| gldm_graylevelnonuniformity | 13691.01 ± 7445.17 | 6873.5 ± 5269.9 | 0.031 |
| gldm_graylevelvariance | 0.05 ± 0.07 | 0.06 ± 0.06 | 0.89 |
| gldm_highgraylevelemphasis | 3.94 ± 0.38 | 3.68 ± 1.10 | 0.46 |
| gldm_largedependenceemphasis | 549.88 ± 43.90 | 499.28 ± 75.19 | 0.07 |
| gldm_largedependencehighgrayleve | 2184.09 ± 221.93 | 1828.5 ± 556.96 | 0.06 |
| gldm_largedependencelowgraylevel | 146.59 ± 27.25 | 170.79 ± 133.65 | 0.55 |
| gldm_lowgraylevelemphasis | 0.28 ± 0.07 | 0.36 ± 0.24 | 0.31 |
| gldm_smalldependenceemphasis | 0.00 ± 0.00 | 0.01 ± 0.00 | 0.13 |
| gldm_smalldependencehighgrayleve | 0.02 ± 0.01 | 0.02 ± 0.03 | 0.48 |
| gldm_smalldependencelowgraylevel | 0.00 ± 0.00 | 0.00 ± 0.00 | 0.11 |
| glrlm_graylevelnonuniformity | 1702.64 ± 605.08 | 1044.4 ± 482.25 | 0.015 |
| glrlm_graylevelnonuniformitynorm | 0.75 ± 0.15 | 0.74 ± 0.18 | 0.92 |
| glrlm_graylevelvariance | 0.13 ± 0.08 | 0.13 ± 0.09 | 0.94 |
| glrlm_highgraylevelrunemphasis | 3.80 ± 0.72 | 3.55 ± 1.30 | 0.58 |
| glrlm_longrunemphasis | 94.35 ± 34.29 | 60.38 ± 37.74 | 0.044 |
| glrlm_longrunhighgraylevelemphas | 375.26 ± 138.55 | 222.21 ± 162.84 | 0.031 |
| glrlm_longrunlowgraylevelemphasi | 24.55 ± 8.26 | 20.12 ± 16.72 | 0.43 |
| glrlm_lowgraylevelrunemphasis | 0.34 ± 0.11 | 0.42 ± 0.24 | 0.34 |
| glrlm_runentropy | 4.15 ± 0.27 | 3.77 ± 0.32 | 0.008 |
| glrlm_runlengthnonuniformity | 294.28 ± 194.43 | 233.78 ± 183.69 | 0.48 |
| glrlm_runlengthnonuniformitynorm | 0.11 ± 0.04 | 0.14 ± 0.06 | 0.19 |
| glrlm_runpercentage | 0.16 ± 0.05 | 0.21 ± 0.08 | 0.08 |
| glrlm_runvariance | 37.93 ± 13.72 | 23.78 ± 15.12 | 0.037 |
| glrlm_shortrunemphasis | 0.27 ± 0.10 | 0.31 ± 0.12 | 0.37 |
| glrlm_shortrunhighgraylevelempha | 0.92 ± 0.49 | 1.10 ± 0.94 | 0.59 |
| glrlm_shortrunlowgraylevelemphas | 0.13 ± 0.09 | 0.16 ± 0.10 | 0.47 |
| glszm_graylevelnonuniformity | 21.67 ± 15.63 | 18.37 ± 13.71 | 0.62 |
| glszm_graylevelnonuniformitynorm | 0.69 ± 0.18 | 0.73 ± 0.14 | 0.63 |
| glszm_graylevelvariance | 0.36 ± 0.33 | 0.26 ± 0.26 | 0.44 |
| glszm_highgraylevelzoneemphasis | 3.27 ± 2.44 | 2.93 ± 2.54 | 0.75 |
| glszm_largeareaemphasis | 8.9e+06 ± 8.7e+06 | 3.9e+0 ± 3.9e+0 | 0.12 |
| glszm_largeareahighgraylevelemph | 3.6e+07 ± 3.5e+07 | 1.3e+0 ± 1.5e+0 | 0.09 |
| glszm_largearealowgraylevelempha | 2.3e+06 ± 2.2e+06 | 1.6e+0 ± 2.3e+0 | 0.48 |
| glszm_lowgraylevelzoneemphasis | 0.69 ± 0.28 | 0.72 ± 0.29 | 0.81 |
| glszm_sizezonenonuniformity | 8.89 ± 6.65 | 6.97 ± 4.67 | 0.47 |
| glszm_sizezonenonuniformitynorma | 0.29 ± 0.11 | 0.34 ± 0.12 | 0.34 |
| glszm_smallareaemphasis | 0.52 ± 0.12 | 0.42 ± 0.22 | 0.23 |
| glszm_smallareahighgraylevelemph | 1.87 ± 1.51 | 1.26 ± 1.51 | 0.37 |
| glszm_smallarealowgraylevelempha | 0.34 ± 0.19 | 0.33 ± 0.24 | 0.84 |
| glszm_zoneentropy | 2.75 ± 0.57 | 2.28 ± 0.81 | 0.13 |
| glszm_zonepercentage | 0.00 ± 0.00 | 0.00 ± 0.00 | 0.07 |
| glszm_zonevariance | 8.5e+06 ± 8.4e+06 | 3.4e+0 ± 3.5e+0 | 0.10 |
| ngtdm_busyness | 109.03 ± 156.94 | 55.35 ± 54.55 | 0.34 |
| ngtdm_coarseness | 0.02 ± 0.03 | 0.03 ± 0.04 | 0.59 |
| ngtdm_complexity | 0.09 ± 0.11 | 0.17 ± 0.32 | 0.45 |
| ngtdm_contrast | 0.00 ± 0.01 | 0.01 ± 0.01 | 0.72 |
| ngtdm_strength | 0.03 ± 0.06 | 0.03 ± 0.04 | 0.99 |

Note.—Data are means ± standard deviations. GLCM = grey level co-occurrence matrix. GLDM = Gray Level Dependence Matrix. GLSZM = Gray Level Size Zone. GLRLM = Gray Level Run Length Matrix. NGTDM = Neighboring Gray Tone Difference Matrix.

## Supplementary Figures


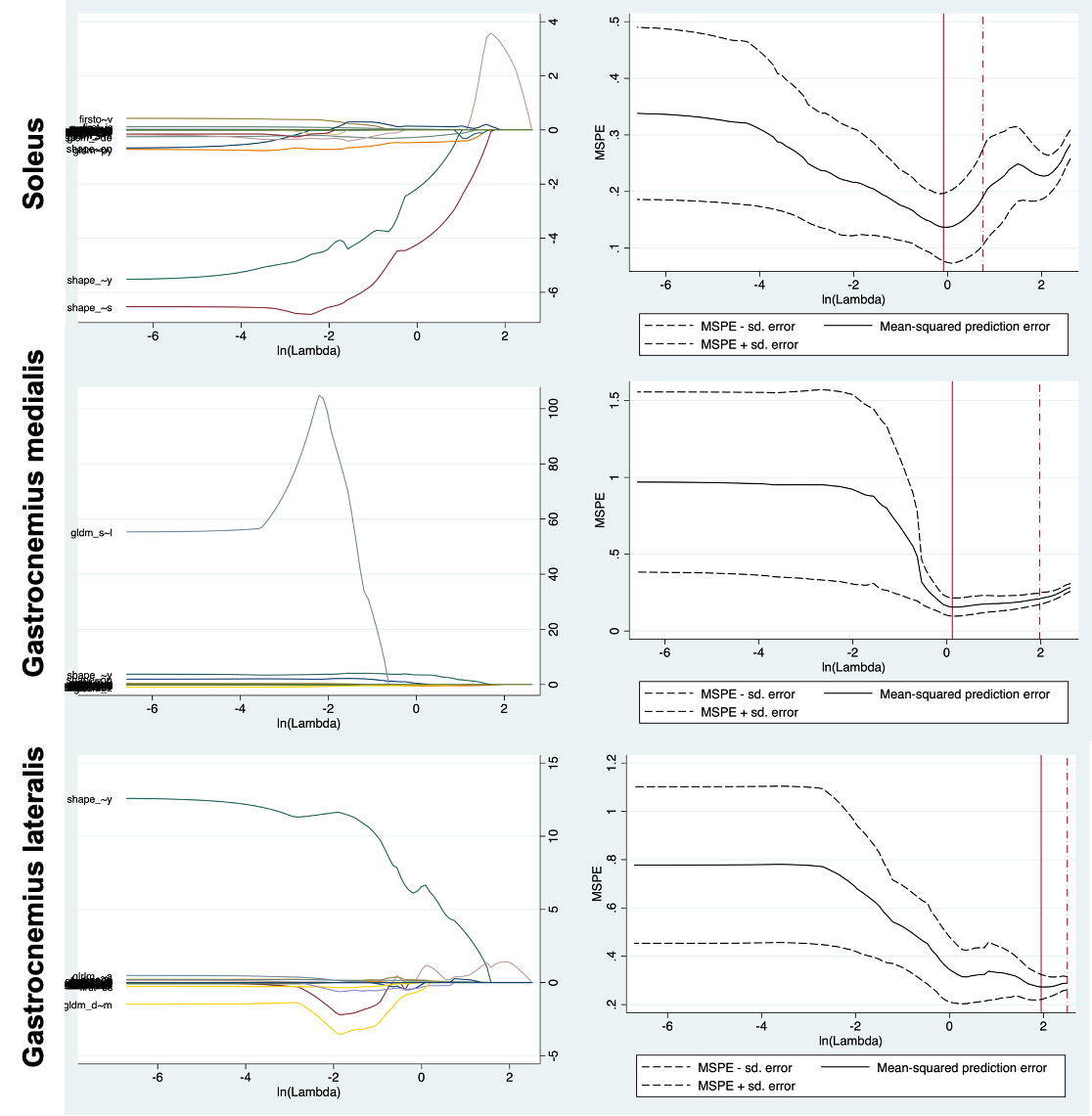


- - 1. **Supplementary Figure 1.** Feature selection using least absolute shrinkage and selection operator (LASSO) model. The partial likelihood deviance was plotted versus log(λ). Tuning parameter (λ) selection in the LASSO model involved the use of 10-fold-cross-validation via minimum criteria. Dotted red vertical lines were drawn at the optimal values by using the minimum criteria and the 1 standard error of the minimum criteria. Optimal λ was around 2 for each model.

~~
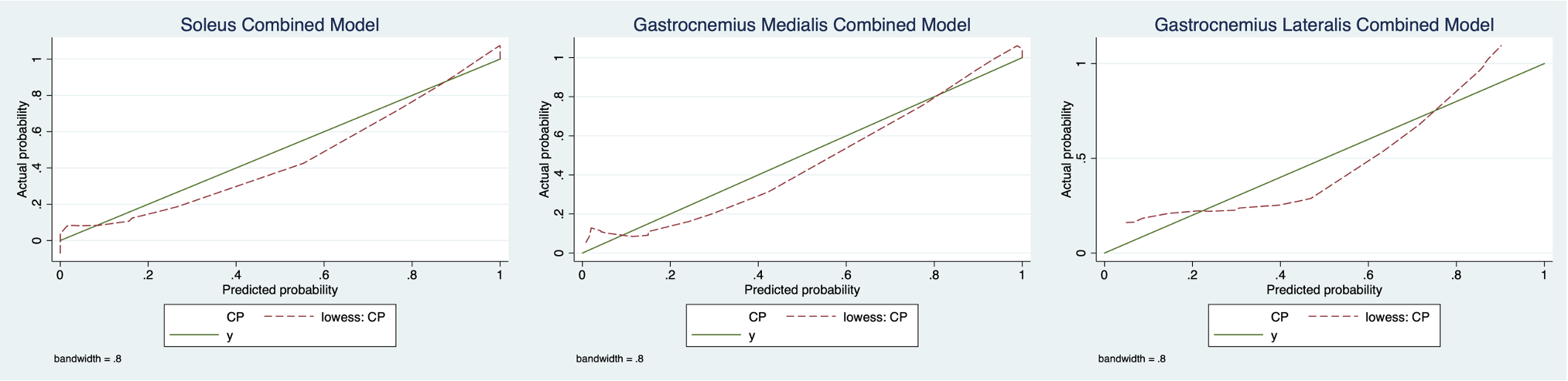
~~

- - 1. **Supplementary Figure 2.** Calibration plots of all combination models for each muscle, which show models calibration in terms of the agreement between the actual and the predicted probability. Predicted probability is plotted on the x-axis; the actual probability is plotted on the y-axis. Diagonal green line = a perfect prediction by an ideal model that corresponds to the actual probability. Dotted red line = performance of the model, a closer lining of which with the diagonal line represents a better prediction.

## References

1. Zwanenburg A, Vallières M, Abdalah MA, Aerts HJWL, Andrearczyk V, Apte A, et al. The Image Biomarker Standardization Initiative: Standardized Quantitative Radiomics for High-Throughput Image-based Phenotyping. *Radiology*. 2020;(5):191145.

2. Van Griethuysen JJM, Fedorov A, Parmar C, Hosny A, Aucoin N, Narayan V, et al. Computational Radiomics System to Decode the Radiographic Phenotype. *Cancer Res*. 2017;77(21):e104–7.

3. Unser M. Sum and Difference Histograms for Texture Classification. *IEEE Trans Pattern Anal Mach Intell*. 1986;PAMI-8(1):118–25.
